# Research on the Mechanism of Calcium-Based Desulfurization Ash Stabilizer for Soft Soil

**DOI:** 10.3390/ma19214626

**Published:** 2026-10-30

**Authors:** Fujia Xu, Fei Gao, Jie Li, Ruiying Wang, Min Pan, Guangchao Wu, Canhua Li

**Affiliations:** 1 School of Metallurgical Engineering, Anhui University of Technology, Ma’anshan 243002, China; 15551768919@163.com (F.X.); yejinlijie@163.com (J.L.); wry201485@163.com (R.W.); w860063878@163.com (G.W.); 2 MCC Huatian Engineering and Technology Corporation, Ma’anshan 243061, China; gaofei4041299@163.com (F.G.); sinopan1985@163.com (M.P.)

**Keywords:** composite stabilizer, soft soil stabilization, mechanical properties, heavy metal concentration assessment

## Abstract

Desulfurization ash is a solid waste generated during the flue gas desulfurization process in the steel industry. Currently, if left untreated, it can pose environmental hazards. The desulfurization ash from semi-dry sintering (SDDA) in this study was used as the primary raw material to prepare a composite stabilizer suitable for soft soil stabilization. The stabilizer formulations with varying SDDA content were designed, and the engineering performance and ecological safety of the stabilized soft soils were comprehensively evaluated. The results indicated that when the SDDA content in the stabilizer was 20%, the 7-day unconfined compressive strength (UCS) reached 0.803 MPa; when the desulfurization ash content was 30%, the 28-day strength reached a maximum of 1.154 MPa. After stabilization, the concentrations of heavy metals such as manganese and chromium in the stabilized soft soil were far below the limits set by relevant national soil pollution risk control standards, indicating that this stabilization system might reduce the migration risk of heavy metals into the environment. Microscopic mechanism analysis revealed that the sulfur-containing components in SDDA promoted the formation of ettringite, while the alkaline environment it provided activated the cementitious system—comprising cement clinker and mineral powder—thereby facilitating the formation of calcium silicate hydrate gel. This study provided a feasible technical approach for the large-scale resource utilization of semi-dry desulfurization ash.

## 1. Introduction

Traditional replacement methods for foundation reinforcement often involve complex construction procedures, high material consumption, and difficulties in achieving effective in situ improvement of soft soils [1]. Cement- and lime-based stabilizers have been widely applied in soft soil stabilization due to their excellent strength development and engineering reliability [2]. However, the production of conventional cementitious materials is associated with high energy consumption and significant CO_2_ emissions, which conflicts with the increasing demand for sustainable and low-carbon infrastructure development [3]. Therefore, the development of environmentally friendly stabilizing materials based on industrial solid wastes has become an important research direction for improving the sustainability of soft soil stabilization technologies [4,5].

Previous studies have demonstrated that the selection and combination of stabilization materials significantly influence the mechanical properties, durability, and environmental performance of treated soils [6,7]. Cement-based materials can provide rapid hydration and early strength development, while supplementary cementitious materials such as fly ash and slag can participate in pozzolanic and latent hydraulic reactions to generate additional calcium silicate hydrate (C-S-H) gels [8]. Chen et al. [9] reported that different cement varieties exhibited different stabilization effects on solidified soils. Chen et al. [10] found that alkali-activated slag binders could effectively stabilize chromium-containing sludge through the formation of hydration products and immobilization of chromium. Tang et al. [11]. demonstrated that fly ash-based stabilization materials improved the strength of contaminated marine sediments mainly through the generation of C-S-H gels. Inorganic stabilization materials containing oxides and aluminosilicate phases can react with water and alkaline substances to form fibrous or network-like hydration structures, thereby enhancing soil strength and reducing pollutant mobility [12]. These studies indicate that industrial solid wastes with reactive mineral components have considerable potential as sustainable alternatives to conventional stabilizers [13].

In recent years, sulfate-rich industrial by-products have attracted increasing attention as potential components of cementitious stabilization systems because of their reactive calcium and sulfur-containing phases [14,15]. Various sulfate-containing wastes, such as desulfurization gypsum and flue gas desulfurization residues, have been investigated due to their ability to participate in hydration reactions and promote the formation of ettringite (AFt) [16]. The formation of ettringite can contribute to pore filling, particle bonding, and early strength development when sufficient calcium and aluminum sources are available [17]. Semi-dry desulfurization ash (SDDA), generated during semi-dry flue gas desulfurization processes in the steel industry, is a typical sulfate-rich industrial by-product containing abundant calcium compounds, calcium sulfite, and calcium sulfate [18]. Owing to the advantages of low investment cost, small land occupation, and relatively simple operation, semi-dry desulfurization technology has been widely adopted in steel industries [19,20]. However, the large-scale generation of SDDA has caused increasing environmental pressure because of its low utilization efficiency and complex chemical characteristics. It has been reported that the annual production of semi-dry desulfurization ash from the steel industry in China reaches approximately 20 million tons [10,17,21,22]. The relatively high Ca/S ratio and the coexistence of multiple calcium-containing phases make the direct utilization of SDDA challenging [23,24]. Therefore, developing efficient approaches for the resource utilization of SDDA is of considerable environmental and engineering significance.

Although previous studies have explored the utilization of desulfurization residues in cementitious materials, several important issues remain unclear: the role of SDDA as a primary component rather than a supplementary additive in complex binder systems; the interaction mechanism between sulfate activation and cementitious gel formation under different SDDA contents; and the engineering applicability and environmental safety of SDDA-based stabilizers. To address these gaps, this study proposes a novel multi-component composite stabilizer integrating SDDA with cement clinker, fly ash, blast furnace slag powder, red mud, and silica fume, aiming to systematically investigate the influence of binder composition on mechanical performance, clarify the coupled hydration mechanisms through XRD, Ftir, and SEM-EDS analyses, and evaluate environmental safety based on heavy metal concentration assessment. This work provides scientific insights into the hydration behavior of complex solid-waste-based binders and practical guidance for low-carbon stabilization technologies in geotechnical engineering.

## 2. Raw Materials and Testing Methods

### 2.1. Raw Materials

The desulfurization ash used in the experiment was taken from the semi-dry sintering desulfurization ash (SDDA) of Maanshan Iron and Steel Group Co., Ltd. Maanshan, China, mainly containing calcium components CaSO_4_ and CaSO_3_∙0.5H_2_O, along with small amounts of CaCO_3_ and Ca(OH)_2_. Other materials included: 42.5 cement clinker (CC) selected from Anhui Conch Cement Co., Ltd. Wuhu, China; Class II fly ash (FA) from Maanshan Power Plant of State Grid Corporation of China, Maanshan, China; Bayer process red mud (RM) from Guangxi Tiangui Aluminum Co., Ltd. Jingxi, China; Level 105 granulated blast furnace slag powder (BGSP) provided by Maanshan Iron and Steel Group Co., Ltd. Maanshan, China; and commercially available silica fume (SF). The chemical compositions of experimental desulfurization ash, cement clinker, fly ash, red mud, mineral powder, and silica fume were analyzed using the XRF method, and the results were shown in Table 1.

**Figure 1:**
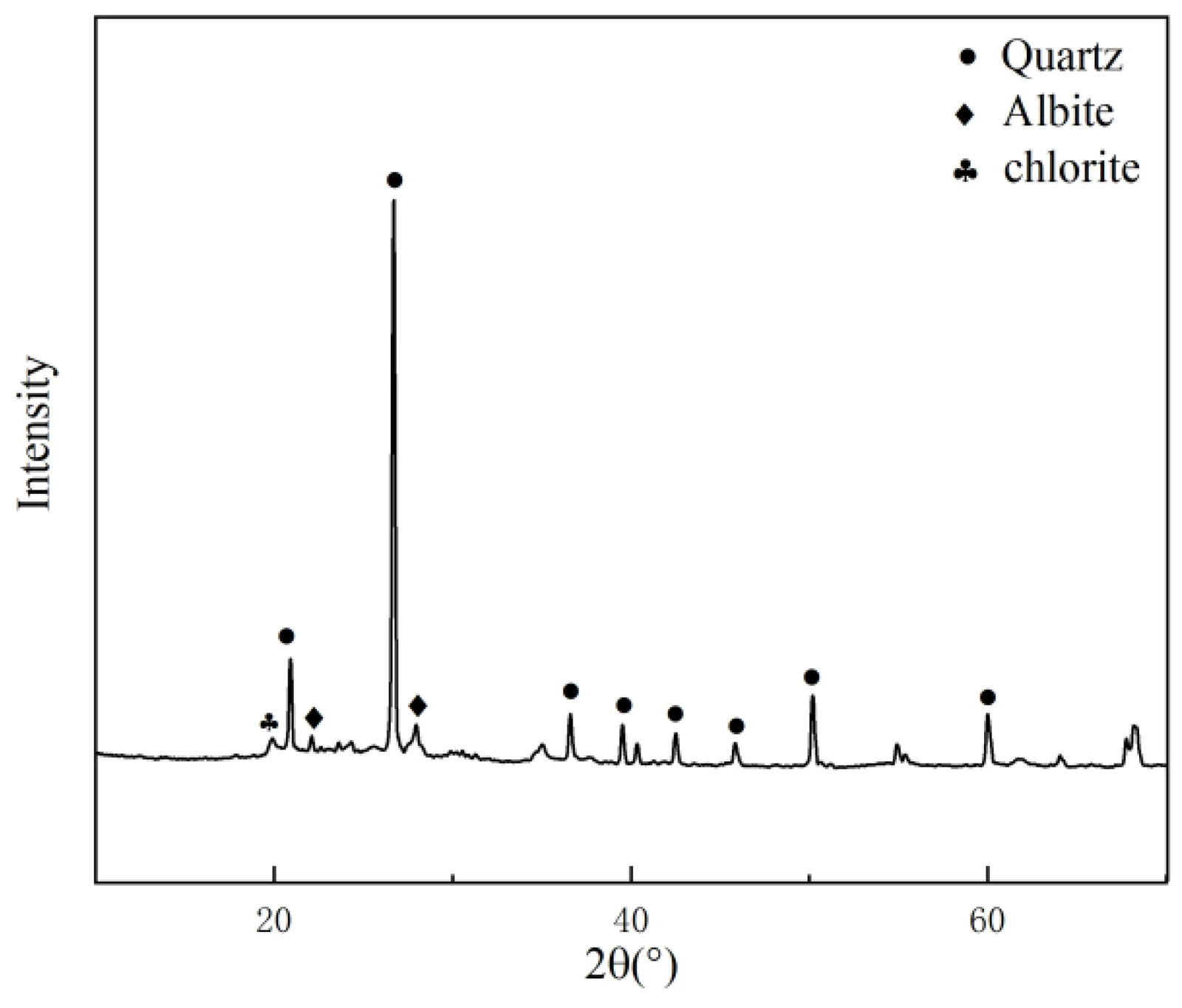
XRD pattern of the river channel stabilized soft soil.

The soft soil used in this test was taken from the riverbed of a planned highway section in He County, Ma’anshan City, Anhui Province, and the stabilized soft soil was generally yellow in color. After collection, the soft soil was sealed and stored in a dry, dark place for subsequent experimental testing. The XRD test of the dried soil was conducted, and the results were shown in Figure 1, indicating that the main mineral components were quartz sand, along with a small amount of aluminosilicate minerals such as albite and chlorite. The main chemical components of the soft soil measured by XRF are shown in Table 2.

The basic physical properties of the stabilized soft soil were determined according to the testing methods in the “Standard for Geo-technical Test Methods” (GB/T50123-2019) [25], and the test results were shown in Table 3.

### 2.2. Experimental Method

The soil sample was dried at 60 °C in a drying oven, ball-milled, and then mixed with an appropriate amount of deionized water to obtain remolded soil with the corresponding water content. According to the mix proportions listed in Table 4, the stabilizer dosage was fixed at 15 wt% of the dry mass of the soft soil. The required amount of oven-dried soil and stabilizer was accurately weighed and thoroughly mixed. Subsequently, deionized water was added to adjust the moisture content of the remolded soil before mixing at low speed for 2 min to prepare a homogeneous stabilized soil slurry. The water content of the remolded soil was adjusted to the natural moisture content of the sampled soft soil (61.1%) before specimen preparation to ensure consistency among all mixtures. The prepared slurry was cast into pre-oiled prism molds with dimensions of 40 × 40 × 160 mm, followed by vibration on a cement mortar vibrating table to eliminate entrapped air. The mold size was selected in accordance with the specimen preparation requirements specified in the adopted testing standard for cement-stabilized materials, ensuring uniform specimen geometry for subsequent mechanical testing. After the specimens were cured for 24 h and demolded, they were placed in a standard curing chamber to cure under conditions of a temperature of 20 ± 2 °C and humidity greater than 95% for 3 days, 7 days, and 28 days to obtain stabilized soil specimens of different ages. Three independent specimens were prepared and tested for each mixture and curing age. For each formulation and curing period, three separate prism molds were individually prepared from independently batched slurries to ensure genuine experimental independence. The mechanical properties were reported as mean values with standard deviations calculated from three replicate measurements. The standard deviation was used to represent experimental variability and was included as error bars in the corresponding figures.

The UCS test was conducted in accordance with the unconfined compressive strength test section specified in the “Test Methods of Soils for Highway Engineering” (JTG 3430-2020) [26]. Prior to testing, the prism specimens were trimmed and ground into cylindrical specimens with a diameter of 40 mm and a height of 100 mm. Before testing, both loading surfaces were carefully polished to ensure uniform contact with the loading platens. The specimens were positioned vertically, with the loading direction along the longitudinal axis of the cylinder. The specimens were loaded under displacement control at a constant loading rate of 1 mm/min until failure. The California Bearing Ratio (CBR) test was conducted according to the CBR test procedure specified in the Standard for Soil Testing of Highway Engineering (JTG 3430-2020). After 28 days of curing, stabilized soil specimens were prepared using the same mixing and moisture adjustment procedures described above. The specimens were compacted under controlled conditions to achieve consistent density among different mixtures. The compaction energy, molding moisture content, and dry density were maintained according to the requirements of JTG 3430-2020. The CBR tests were conducted under unsoaked conditions, and three independent specimens were prepared and tested for each formulation. The total concentrations of heavy metal elements in the stabilized soil samples were determined according to the Standard ‘Microwave digestion method for the total content of metal elements in soil and sediment’ (HJ832-2017) [27]. This method was used to characterize the total concentrations of selected heavy metals rather than their leaching behavior or environmental mobility. To investigate the performance of the SDDA-based composite stabilizer under different formulation conditions, nine mix designs were prepared as shown in Table 4. The purpose of the experimental design was to optimize the overall performance of the multi-component stabilizer rather than to evaluate the isolated effect of SDDA content. The SDDA content was adjusted within the composite binder system, while the proportions of cement clinker, fly ash, blast furnace slag powder, and silica fume were simultaneously modified to maintain the balance between sulfate activation and cementitious phase formation.

The variation in binder composition was designed based on the complementary functions of different components. SDDA provided calcium and sulfate sources for ettringite formation, cement clinker contributed to early hydration and strength development, while fly ash, slag powder, and silica fume promoted long-term pozzolanic reactions and C-S-H gel formation. Therefore, the mechanical performance of each mixture should be considered as the combined result of SDDA incorporation and the synergistic effects among different binder components.

## 3. Results and Discussion

Based on existing studies, desulfurization ash can react with the alumina in cement clinker or mineral powder to form ettringite, which improves the strength of the samples. Cement clinker and slag powder provide the core cementitious phases for the system. Red mud can provide an alkaline environment for stabilization or serve as filler. Fly ash and silica fume can improve long-term strength through pozzolanic reactions, and their fine particles can fill pores and make the structure denser. All mechanical properties presented in this section are expressed as mean ± standard deviation based on three independent specimens. Error bars in the figures represent the standard deviation among replicate specimens. The observed differences among formulations should therefore be interpreted considering both the mean performance and experimental variability.

### 3.1. Performance Analysis Results

#### 3.1.1. Unconfined Compressive Strength

**Figure 2:**
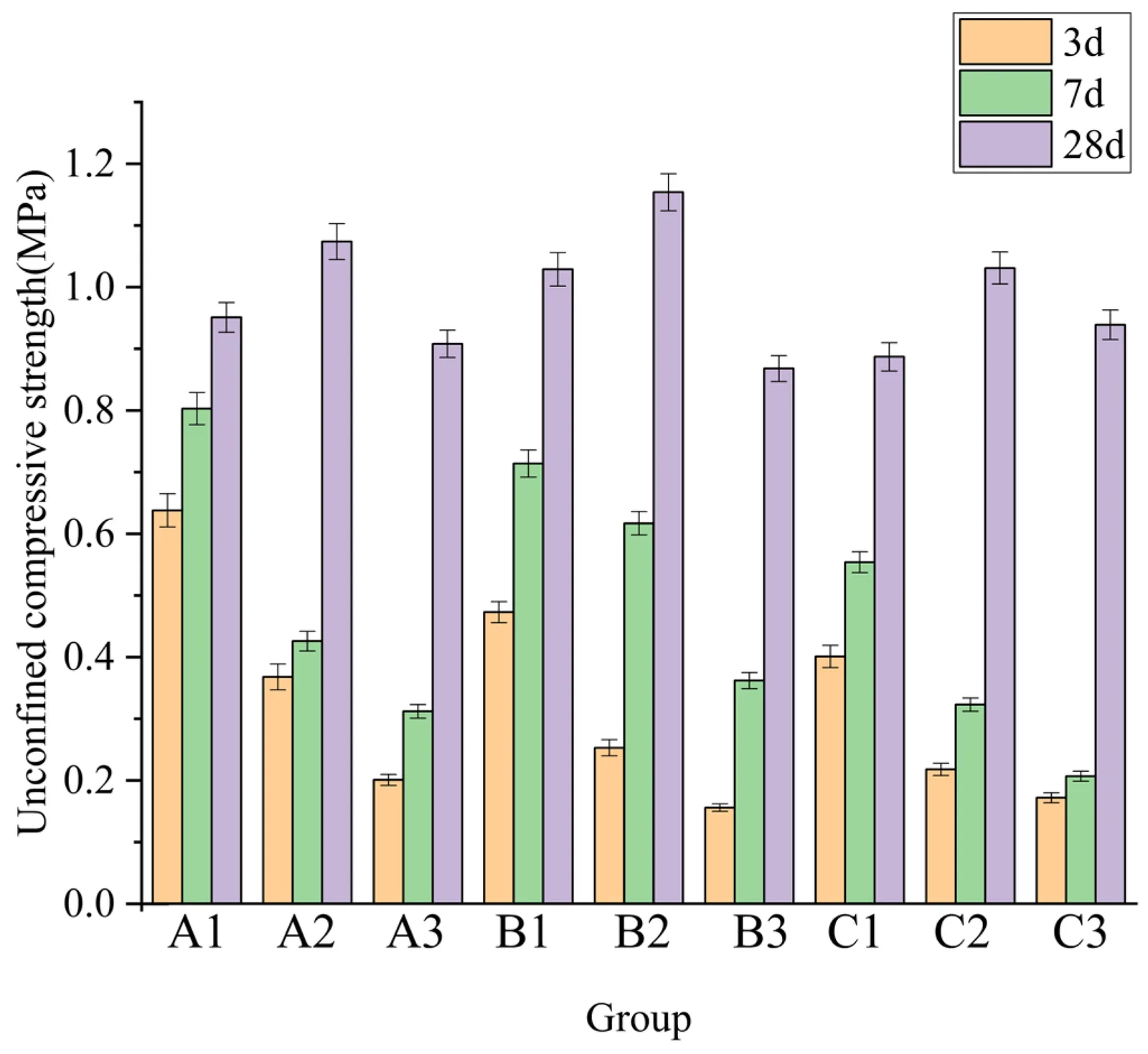
Stabilized soft soil’s strength at 3 d, 7 d, and 28 d.

The UCS of stabilized soft soil cured for 3 and 7 days is shown in Figure 2. For mixtures with the same SDDA content, increasing the cement clinker proportion enhanced the early compressive strength because additional hydraulic phases promoted hydration and C-S-H gel formation. For example, within Group A (20% SDDA), the 7-day strength of specimen A1 containing 40% cement clinker reached 0.803 MPa, which was higher than those of A2 and A3. However, comparisons among Groups A, B, and C should be interpreted with caution because the increase in SDDA content was accompanied by simultaneous adjustments in the proportions of fly ash, blast furnace slag powder, and silica fume. Therefore, the differences in strength among these groups reflect the combined effects of SDDA incorporation and the overall binder composition rather than the independent influence of SDDA alone. A higher content of desulfurization ash may slow down the hydration process of cement clinker. In the cementitious system, the hydration of cement clinker was faster than that of fly ash or slag powder, and its contribution to early strength development was greater than that of slag and fly ash. In the early stage, the rapid hydration of cement clinker provided CH and early C-S-H. The micro-filler effect and high pozzolanic activity of silica fume can accelerate the hydration reaction, while red mud provided an alkaline environment. In contrast, the hydration process of slag powder was slower, which could slowly increase strength and improve durability.

The 28-day UCS results of the stabilized soil are shown in Figure 2. Unlike the early-age strength, no simple monotonic trend was observed at 28 days, indicating that long-term strength development was governed by the interaction of multiple hydration reactions. At this stage, the contribution of cement clinker hydration gradually decreased, whereas the pozzolanic reactions of fly ash and blast furnace slag powder became increasingly significant. The calcium hydroxide released from cement clinker hydration activated the latent hydraulic activity of fly ash and slag powder, promoting the continuous formation of C-S-H gel and improving the compactness of the stabilized soil matrix.

Meanwhile, SDDA supplied sulfate ions that reacted with calcium aluminate phases to generate ettringite (AFt). Moderate AFt formation effectively filled internal pores and enhanced particle bonding. However, excessive SDDA incorporation inevitably reduced the proportion of other reactive cementitious materials in the composite binder, limiting the generation of C-S-H gel despite the increased sulfate availability. Consequently, the long-term strength was determined by the balance between AFt formation and cementitious gel development rather than by the SDDA content alone.

This synergistic mechanism explains why mixtures containing 30% cement clinker (A2, B2, and C2) exhibited relatively high 28-day strengths of 1.074 MPa, 1.154 MPa, and 1.031 MPa, respectively. At this clinker dosage, sufficient calcium hydroxide was available to sustain the pozzolanic reactions of fly ash and slag powder while maintaining adequate sulfate activation for AFt formation. In contrast, excessive clinker replacement by SDDA or insufficient reactive supplementary cementitious materials weakened the balance between hydration and sulfate activation, resulting in relatively lower long-term strength. All mixtures with 30% cement clinker exceeded the minimum compressive strength requirement of 0.8 MPa for highway base materials specified in JTG/T F20—2015.

These results indicate that optimizing the proportion of individual binder constituents is more important than simply increasing the SDDA dosage, highlighting the importance of balancing hydraulic hydration, pozzolanic reactions, and sulfate activation in the composite stabilizer system.

#### 3.1.2. CBR

**Figure 3:**
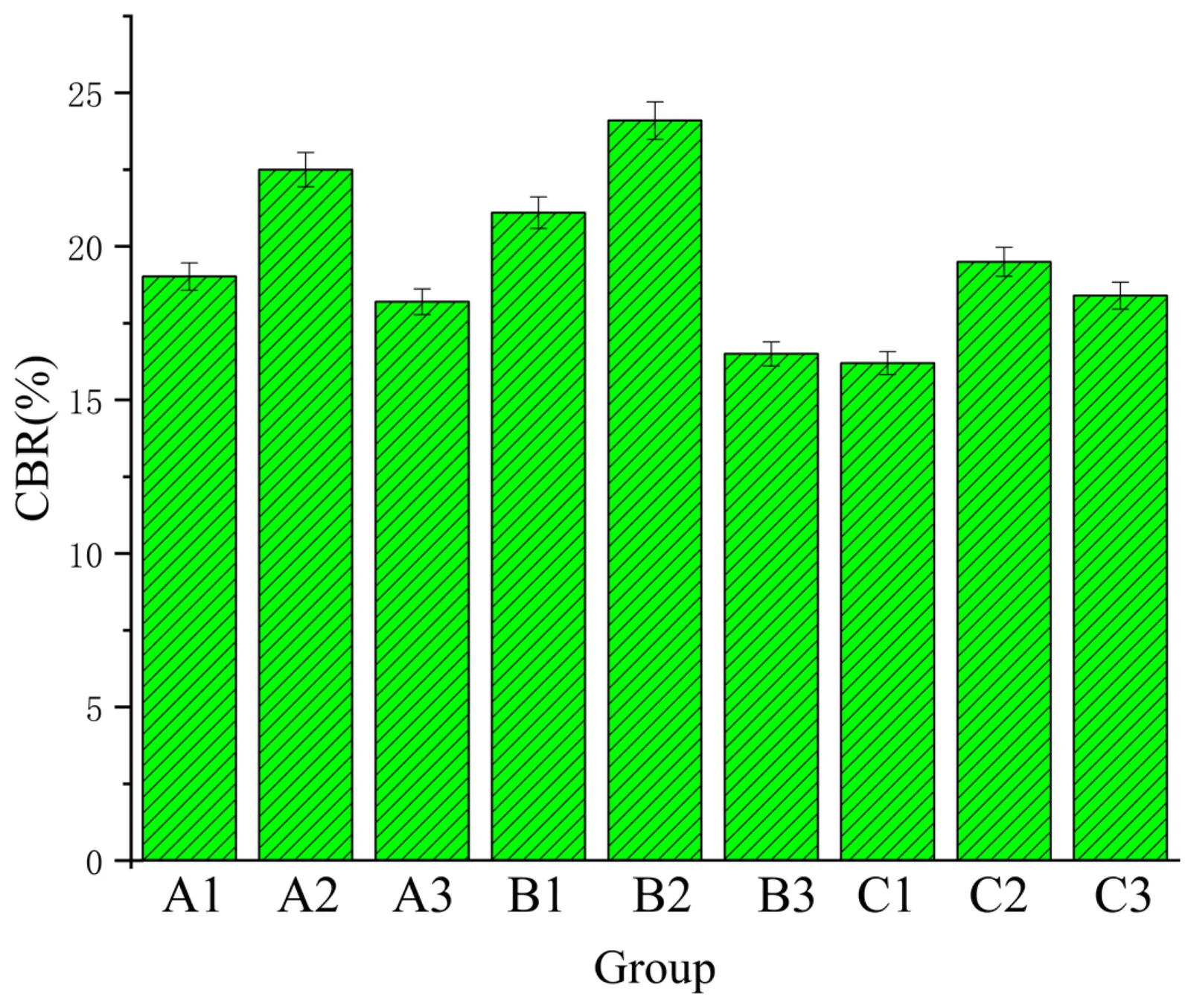
CBR of the stabilized soft soil.

The CBR value can reflect the ability of the soil to resist local shear failure under its self-weight and load and was the control for the strength of soil used in embankment filling and the evaluation index for road performance specified in current roadbed design codes. The CBR test was conducted after 28 days of curing of the stabilized soft soils. Figure 3 showed the CBR test results of different stabilized soft soils. The overall trend of CBR change in stabilized soft soils was similar to the change in stabilized soft soil strength. The principle of the stabilizer improving the CBR of the soil was similar to the principle of improving the strength of the soil. The cementitious phases fully connected the soil particles and filled their pores, thereby forming a solid skeletal network. After a long period of curing, it improved the compressive strength of the soil and also significantly enhanced deformation resistance. The supporting effect of the solid skeletal network significantly reduced the plastic deformation of the soil under load, thereby improving the CBR of the soil.

A comparison of the 28-day UCS and CBR values across all formulations further confirmed the consistency between these two mechanical indicators. Test specimens exhibiting higher 28-day UCS generally corresponded to higher CBR values, demonstrating a positive correlation between the two parameters. For example, specimen B2, which achieved the highest 28-day UCS of 1.154 MPa among all mixtures, also exhibited one of the highest CBR values of 24.1% in Group B. Similarly, specimen A1 showed a 7-day UCS of 0.803 MPa and a 28-day CBR of 19.02%, while specimen C3 with the highest SDDA content (60%) exhibited relatively lower values of both UCS (0.939 MPa) and CBR (18.4%). This consistent trend suggests that the hydration products governing strength development—namely C-S-H gel and ettringite—simultaneously contribute to resistance against localized penetration deformation. The formation of a dense skeletal network through these hydration products benefits both unconfined compressive resistance and bearing capacity under dynamic loading.

#### 3.1.3. Heavy Metal Detection

The cured soil samples A1 and C3, with the lowest and highest desulfurization ash content, respectively, were tested for heavy metals such as Mn, Zn, Cd, Pb, and Cr after stabilization, as shown in Table 5. The cured soil samples A1 and C3, representing the lowest and highest SDDA contents, respectively, were analyzed for the total concentrations of Mn, Zn, Cd, Pb, and Cr. Compared with untreated soft soil, the total concentrations of Mn and Cr decreased after stabilization. However, these results only reflect changes in total heavy metal concentrations and cannot directly demonstrate reduced leaching potential or immobilization efficiency. The observed decrease may be partially associated with the dilution effect caused by the addition of the stabilizer. The measured total concentrations of heavy metals after stabilization were below the corresponding screening values specified in the Standard ‘Soil Environmental Quality—Risk Control Standard for Soil Contamination of Development Land’ (GB 36600-2018). These results indicate that the total heavy metal concentrations of the investigated samples meet the referenced criteria; however, further leaching tests are required to evaluate the actual environmental mobility and long-term release risk of heavy metals. The total concentrations of heavy metals in the C3 group were below the referenced control limits, indicating that the measured metal concentrations satisfied the specified criteria. However, additional leaching tests are necessary before making conclusions regarding long-term environmental suitability.

### 3.2. Mechanism Analysis

To further reveal the mechanism of calcium-based desulfurization ash curing-stabilized soft soil, the XRD, Ftir, and SEM characterization methods were employed to investigate the mineral composition, phase characteristics, and microstructure evolution of selected cured specimens. Specimens A1 (20% SDDA, 40% CC) and C2 (50% SDDA, 30% CC) were selected as representative formulations for microstructural characterization because they correspond to the lowest and a relatively high SDDA content in the experimental matrix, respectively, thereby enabling a comparison of hydration products under contrasting sulfate availability conditions. A1 achieved the highest 7-day UCS among all mixtures, while C2 exhibited one of the highest 28-day strengths among the high-SDDA groups, making these two specimens mechanically representative of the overall dataset. It should be noted that the microstructural observations are not intended to represent every individual mixture in the full experimental matrix; rather, they are intended to illustrate the dominant hydration mechanisms governing the two contrasting compositional regimes—low and high SDDA incorporation—that collectively explain the strength trends observed across all groups. SEM-EDS analysis was conducted on specimen C2 to characterize the microstructural features and elemental compositions of selected regions under high sulfate availability, as this condition is most relevant to understanding the interplay between ettringite formation and C-S-H gel development.

#### 3.2.1. XRD Analysis

**Figure 4:**
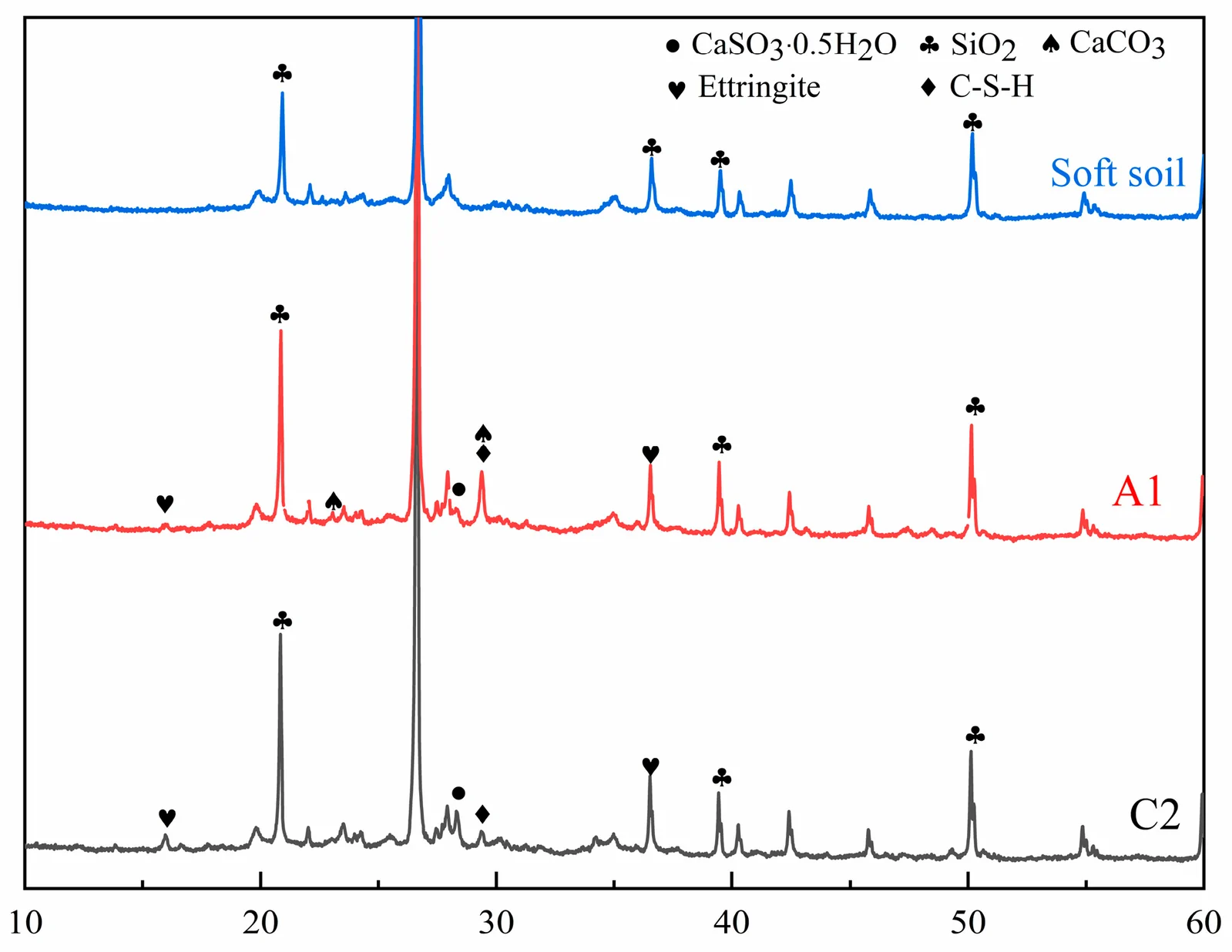
XRD patterns of stabilized soft soil specimen.

Figure 4 shows the XRD patterns of the desulfurization ash-stabilized soil of groups A1 (20% SDDA) and C2 (50% SDDA) after 28 days of curing, representing low and high SDDA incorporation levels, respectively. By comparison with the untreated stabilized soft soil, the diffraction peaks of crystalline phases, including AFt and CaSO_3_·0.5H_2_O, can be observed in both stabilized soil specimens. In addition, a broad and diffuse scattering feature in the range of approximately 25–35° 2θ was detected, which is typically attributed to the poorly crystalline or amorphous nature of C-S-H gel [2]. Although C-S-H cannot be unambiguously identified by its sharp diffraction peaks due to its characteristically low crystallinity, its formation was further confirmed by the Si-O-Si absorption bands at approximately 540 cm^−1^ and 1037 cm^−1^ in the Ftir spectra and the Ca- and Si-enriched amorphous regions observed in SEM-EDS analysis. These combined results confirm that the incorporation of SDDA-based composite stabilizer promotes the formation of these hydration products regardless of SDDA dosage. The contrast between A1 and C2 further allows the relative intensity changes in ettringite diffraction peaks and the degree of the broad amorphous hump associated with C-S-H gel to be correlated with the variation in sulfate availability across the experimental matrix.

The volume of ettringite was much larger than the volume of the substances before the reaction. The formation of ettringite filled the gaps between particles, and excessive formation can also cause the volume of stabilized soft soil to expand. In the early stage of stabilization, a large amount of ettringite filled the internal pores and cracks of the solidified body, making the internal structure denser. However, the formation of ettringite alone cannot link all parts into a solid overall structure. The gel substances generated by the hydration of cementitious materials such as cement clinker played a crucial role in this process; the interstructure formed by ettringite and the gel substances created a polymer-like structure, thereby achieving the stabilization effect. In addition, with increasing SDDA incorporation, the diffraction peak intensity of ettringite became more pronounced because the higher sulfate availability promoted AFt formation. However, the increase in SDDA content was accompanied by simultaneous adjustments in the proportions of other cementitious materials. Consequently, the reduced availability of reactive aluminosilicate components limited the formation of C-S-H gel, suggesting that the hydration products resulted from the synergistic interaction of all binder constituents rather than from SDDA alone. Although a large amount of ettringite can effectively fill the pores, the lack of binding from the gel substance prevented the solidified body from forming a highly bonded whole, leading to a decrease in strength. Furthermore, the excessive ettringite, due to its own expansion effect, squeezed the soil body, which instead led to the formation of cracks, exerting a negative impact on the strength.

In addition, when calcium sulfate was not in excess, the excess metastable calcium aluminate hydrate (C-A-H) formed by hydration of tricalcium aluminate (C_3_A) in cement could not be converted into ettringite and instead converted into calcium silicate aluminate hydrate (C-A-S-H). Furthermore, characteristic peaks of CaCO_3_ and calcium sulfite hemihydrate were detected in the cured stabilized soft soil. CaCO_3_ was chemically relatively stable and originated from unreacted materials or the partial stabilization of CO_2_ from the air during the hydration process; calcium sulfite hemihydrate was an inert chemical substance that was insoluble in water and could not provide sulfate ions during the hydration process to promote the formation of ettringite.

#### 3.2.2. Ftir Analysis

**Figure 5:**
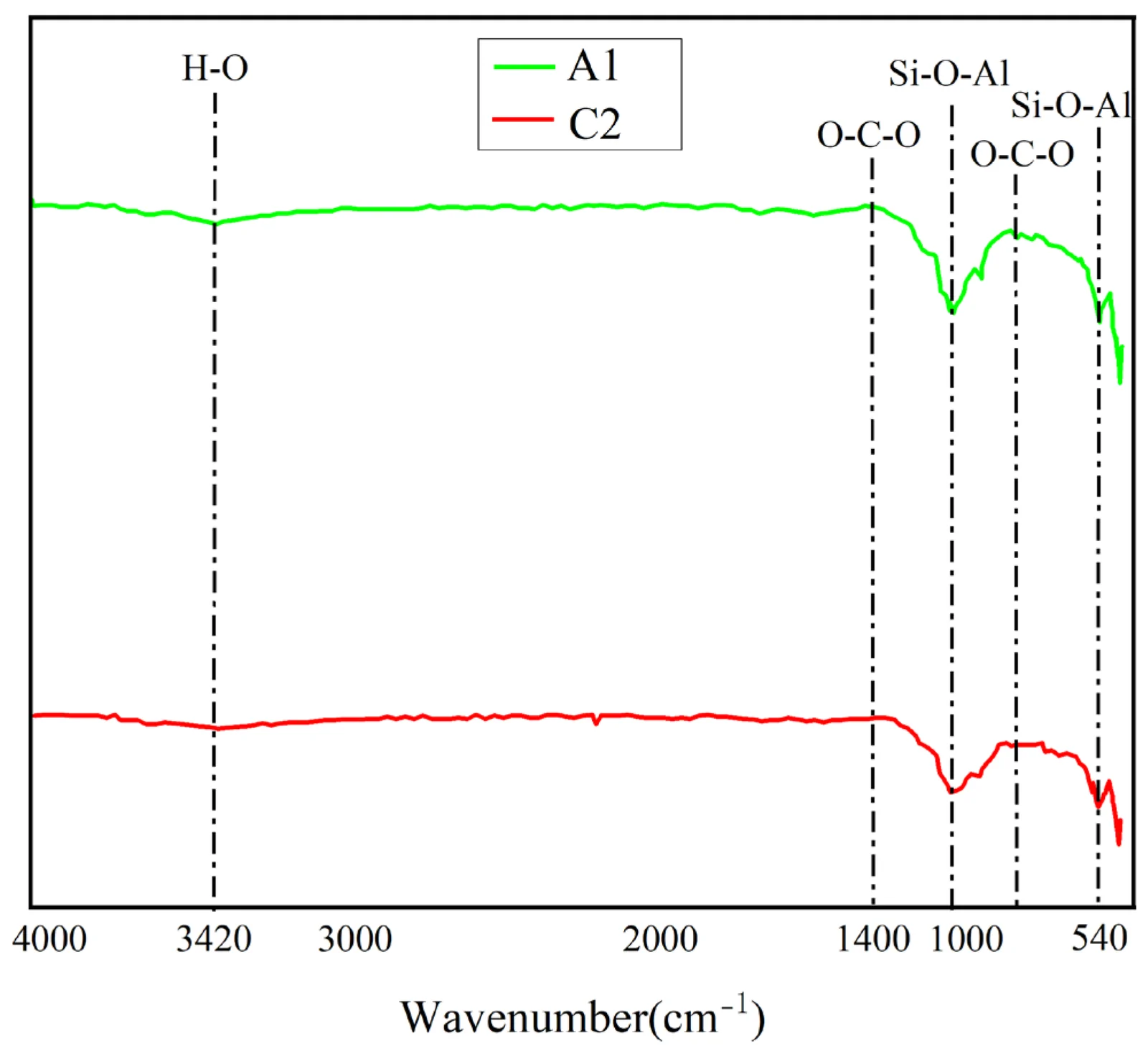
Ftir spectra of cured soil for groups A1 and C2.

Figure 5 showed the Ftir curves of the two groups of stabilized soil after 28 days of curing. As shown in Figure 5, a broad absorption band observed near 3420 cm^−1^ corresponded to the O-H stretching vibration and H-O-H bending vibration of water molecules, which may be caused by the water molecules in the C-S-H gel of the specimens. The absorption peak of group A1 at this position was more prominent than that of group C2, indicating a higher degree of hydration reaction and the generation of more C-S-H gel, which was consistent with the peak patterns in XRD. The absorption bands located at approximately 540 cm^−1^ and 1037 cm^−1^ corresponded to the Si-O-Si vibrations in the C-S-H gel. This was due to the synergistic effect of the co-incorporation of desulfurization ash and the cementitious system and the formation of C-S-H gel. The characteristic peaks located at 1400 cm^−1^ and 900 cm^−1^ were induced by the asymmetric stretching vibration of the O-C-O bond of the CO_3_^2−^ ion. This characteristic peak indicated that a substance reacting to form O-C-O bonds exists in the samples, which may be due to the combination of CO_2_ in the air with calcium ions during the hydration process to form CaCO_3_ crystals, consistent with the characteristic peaks of CaCO_3_ crystals detected in the analysis of Figure 4. However, the CaCO_3_ crystals generated by carbon fixation would have a certain impact on the cementitious phase, which may affect the continuity of the cement products and be detrimental to the long-term stability of the stabilized soft soil. Therefore, exploring the proportion that was conducive to alleviating the carbonation of all-solid cementitious materials and reducing the calcium sulfite hemihydrate in desulfurization ash was a future research direction.

#### 3.2.3. Sem Analysis

**Figure 6:**
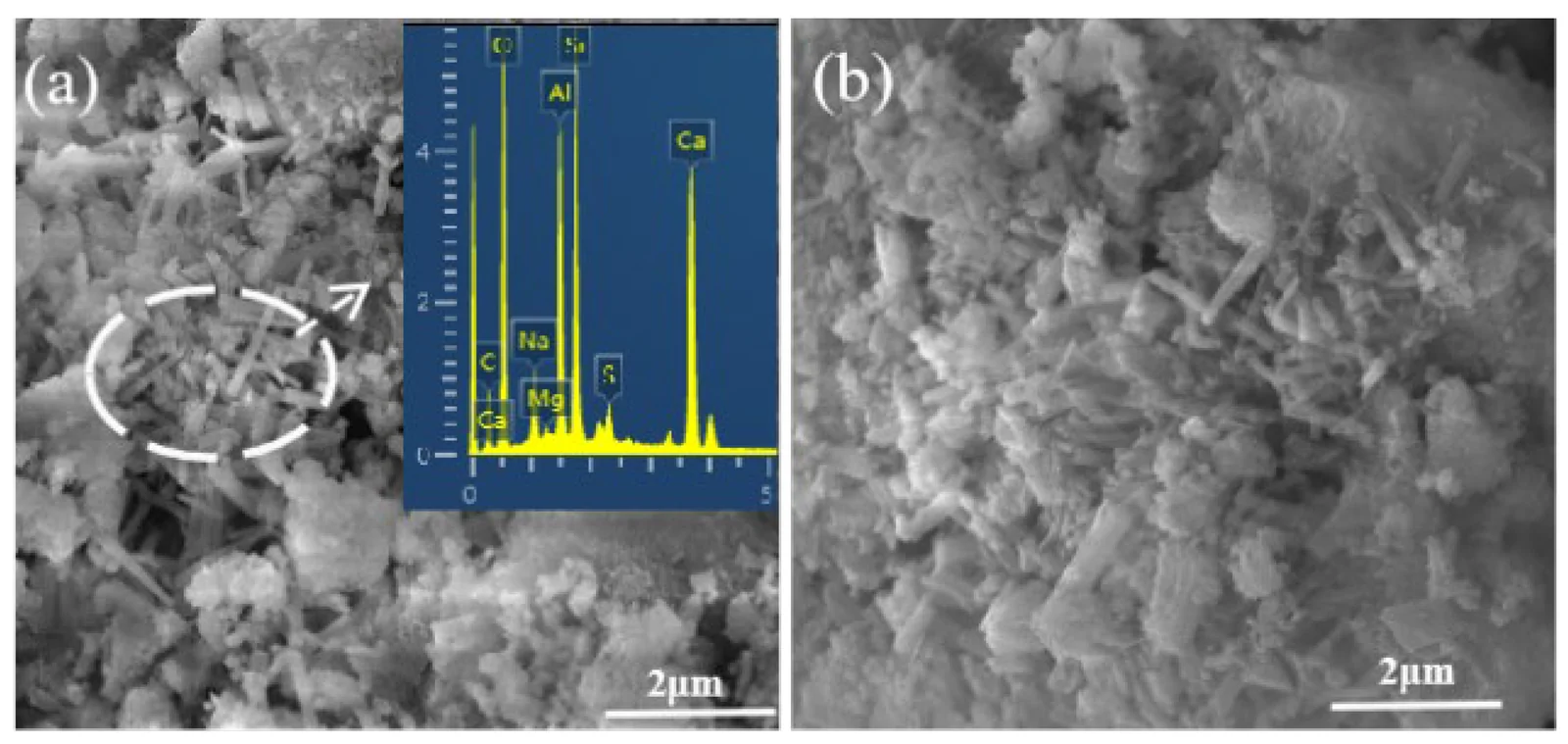
SEM image and representative EDS spectrum of soil specimen stabilized with 15 wt% C2 composite stabilizer (50% SDDA, 30% CC). (**a**,**b**) represent different morphological features of the same set of samples.

The microstructure of the C2 group cured soil specimen (50% SDDA, 30% CC) was characterized by SEM coupled with EDS analysis, and representative images are presented in Figure 6. Considering that the C2 mixture exhibited relatively high 28-day strength among the high-SDDA formulations, it was selected to investigate the hydration products under conditions with relatively high sulfate availability. The SEM observations were used to identify morphological features, while the EDS elemental distributions and point analyses were combined with XRD and Ftir results to assist phase interpretation. Therefore, the identification of hydration products was based on multiple characterization techniques rather than morphology or elemental composition alone. As shown in Figure 6, the SEM images reveal that the soil particles were partially covered by newly formed hydration products. Needle-like crystals and amorphous gel-like regions were observed in the selected areas. The EDS spectrum collected from the representative analysis region confirmed the co-presence of Ca, Si, Al, S, and O signals within the observed microstructure, indicating the coexistence of multiple hydration product phases. The identification of ettringite and C-S-H phases relies primarily on a combined interpretation of XRD diffraction patterns and Ftir absorption spectra, while SEM observations provide supporting morphological evidence.

The needle-like crystals observed in SEM images are consistent with the typical morphology reported for ettringite; however, morphology alone is insufficient for phase identification. The co-detection of Ca, Al, and S signals in the EDS spectrum, together with the characteristic AFt diffraction peaks detected by XRD and the corresponding infrared absorption features identified by Ftir, collectively support the interpretation that ettringite is among the hydration products present. Similarly, the amorphous gel-like regions are interpreted as C-S-H gel based on the combined morphological evidence from SEM observations and the characteristic Si-O-Si absorption bands detected by Ftir at approximately 540 cm^−1^ and 1037 cm^−1^, as well as the broad amorphous hump in the XRD patterns [28]. Furthermore, the identification results were supported by XRD and Ftir data. The ettringite crystals and C-S-H gel substances generated in the system can fill the large pores inside the soil and further tightly connect each part to dense aggregates, thereby significantly improving the density of the matrix.

It should be noted that the present experimental program was designed to optimize the formulation of a multi-component SDDA-based composite stabilizer rather than to isolate the independent effect of SDDA content. Because the proportions of other cementitious constituents were adjusted simultaneously to maintain the functionality of the binder system, the contribution of SDDA cannot be completely separated from the synergistic effects of the composite formulation. Future studies employing single-variable experimental designs are needed to quantify the independent role of SDDA in the stabilization process.

## 4. Conclusions

Taking stabilized soft soil from a highway section in Anhui Province, China, as the research object, this study experimentally evaluated the mechanical properties, heavy metal concentrations, and hydration characteristics of SDDA-based composite stabilizer-treated soils. The main experimental findings and the proposed hydration mechanisms are summarized as follows:

(1) The mechanical properties of stabilized soft soil were jointly affected by the SDDA dosage and the proportions of other cementitious materials in the composite stabilizer. An appropriate balance between sulfate-rich SDDA and hydraulic cementitious components was essential for achieving satisfactory mechanical performance. Among the investigated mixtures, specimen A1 containing 20% SDDA achieved a 7-day UCS of 0.803 MPa, while mixtures containing 30% cement clinker exhibited the highest 28-day strength.

(2) The incorporation of SDDA-based stabilizer decreased the measured total concentrations of certain heavy metals, particularly Mn and Cr, in the stabilized soil samples. The detected total concentrations of heavy metals were below the corresponding screening values specified in GB 36600-2018. However, these results only demonstrate the change in total heavy metal concentrations after stabilization and cannot directly verify the reduction in heavy metal migration or leaching risk. Additional leaching tests are required to evaluate the long-term environmental mobility and release behavior of heavy metals.

(3) XRD, Ftir, and SEM-EDS analyses provided complementary evidence for the formation of hydration products, including C-S-H gel and ettringite (AFt) in the stabilized soil specimens. The phase identification was based on the combined interpretation of diffraction characteristics, infrared vibration features, EDS elemental signals, and microstructural morphology observations. These products were considered to contribute to matrix densification and strength development through pore filling and particle bonding. The observed microstructural evolution suggests that the synergistic hydration reactions among SDDA and other cementitious components played an important role in improving the mechanical performance of stabilized soil.

## Figures and Tables

**Table 1:** Main chemical constituents of the main raw materials.

w/%	CaO	SO_3_	Cl	Fe_2_O_3_	K_2_O	MgO	SiO_2_	TiO_2_	Na_2_O	Al_2_O_3_	Others
SDDA	55.49	31.33	4.10	3.47	1.82	0.87	0.82	0.59	0.51	0.49	0.51
CC	45.18	3.10		4.01	0.53	4.55	21.93			12.52	
FA	4.52	1.04		4.46	1.79	1.61	52.71		1.22	30.44	
SF	0.83	0.74		0.82	5.56	5.93	83.14		1.38		
RM	25.04			30.76	1.18		17.25	5.14	1.08	19.55	
BGSP	38.14	1.80		0.38	0.34	8.33	32.53	0.98	0.64	16.41	0.27

**Table 2:** Main chemical constituents of the soft soil in river channels.

	SiO_2_	Al_2_O_3_	Fe_2_O_3_	K_2_O	MgO	TiO_2_	Na_2_O	CaO
Chemical composition (w/%)	68.30	15.60	7.18	2.92	1.31	1.22	0.99	0.88

**Table 3:** Basic physical properties of the river soft soil.

Moisture Content (w/%)	Liquid Limit (w/%)	Plastic Limit (w/%)	Plasticity Index	Liquidity Index	Organic Matter (w/%)
61.1	47.83	28.46	19.37	1.69	1.76

**Table 4:** Mix design of desulfurization ash-based stabilizer.

Sample No.	Composition (wt%)					
	SDDA	CC	FA	BGSP	RM	SF
A1	20	40	10	15	5	10
A2	20	30	15	20	5	10
A3	20	20	20	25	5	10
B1	30	40	5	10	5	10
B2	30	30	10	15	5	10
B3	30	20	15	20	5	10
C1	40	40	-		5	15
C2	50	30	-		5	15
C3	60	20	-		5	15

**Table 5:** Total concentrations of heavy metals in stabilized soft soil after stabilization (mg/kg).

	Mn	Zn	Cd	Pb	Cr
A1 group cured, stabilized soft soil with 20% desulfurization ash	2.78	0.29	/	0.42	0.51
C3 group cured, stabilized soft soil with 60% desulfurization ash	3.16	0.34	/	0.54	0.40
Unconsolidated stabilized soft soil	10.31	2.38	/	0.63	1.87
First-class construction land screening value	-	-	20	400	-
Second-class construction land screening value	-	-	65	800	-

‘/’: Cadmium (Cd) concentration is below the detection limit of 0.02 mg/kg.

## Data Availability

The datasets generated and analyzed during the current study, including mechanical property measurements, heavy metal concentration results, and microstructural characterization data, are available from the corresponding author upon reasonable request.
